# The consequences of valproate exposure in utero

**DOI:** 10.1007/s00415-016-8269-y

**Published:** 2016-08-26

**Authors:** Rhys H. Thomas, Neil P. Robertson

**Affiliations:** Institute of Psychological Medicine and Clinical Neuroscience, University Hospital of Wales, Cardiff University, Heath Park, Cardiff, CF14 4XN UK

## Introduction

Prospective studies have unequivocally identified that valproate poses an unacceptable risk to the foetus in utero, prompting European and national bodies to issue unambiguous advice to neurologists. In 2014, The European Medicines Agency recommended strengthening the restrictions on the use of valproate in women and girls. The joint task force of International League Against Epilepsy-commission on European affairs and European Academy of Neurology responded with a detailed letter, later published in Epilepsia, which included seven recommendations. The first of which is “Where possible, valproate should be avoided in women of childbearing potential.” In January 2015, the Medicines and Healthcare products Regulatory Agency (MHRA) in the United Kingdom issued stringent guidance regarding the prescription of valproate to women of child-bearing potential. The data supporting these statements were based on robust prospective research approximating the risk of major malformations at one in ten, and that four in ten children are at risk of neurodevelopmental disorders. The advice is, therefore, that first valproate should not be used in girls or women of child-bearing potential unless other treatments are ineffective or not tolerated, and second that women of child-bearing potential must use effective contraception during treatment.

However, valproate cannot simply be discarded from the panoply of anti-epileptic medications since it remains one of the most efficacious therapies; especially for those patients with genetic generalised epilepsy. In recognition of this recent MHRA guidance states “No-one should stop taking valproate without discussing it first with their doctor and the benefits of valproate treatment must be carefully balanced against the risks.” However, despite the advice offered, there remains much that we do not know about valproate and foetal outcomes. Below we discuss three papers that aim to improve the description of risks to the foetus of valproate exposure including foetal loss, autism spectrum disorder and congenital malformations.

## Dose-dependent teratogenicity of valproate in mono- and polytherapy: an observational study

A consistent finding from epilepsy and pregnancy registries is higher doses of valproate are less safe than lower doses of valproate, and that valproate used in combination with other drugs is more cognitively and physically teratogenic than when used alone. For women with genetic generalised epilepsy who need valproate for adequate seizure control consideration should, therefore, be given as to whether a minimum valproate dose together with an alternative concomitant therapy or the smallest efficacious dose of valproate in monotherapy should be used. The International Registry of AEDs and Pregnancy (EURAP) is an observational study established in 1999 that brings together investigators from 42 countries from Europe, Asia, Australia, and Latin America. It primarily studies major congenital malformations by recruiting women prior to week 16 of pregnancy. As such, EURAP studies can often provide the largest sample seizes for sub-group comparison studies. This study identified 1224 pregnancies with valproate as monotherapy and 364 when valproate was taken with another anti-epileptic drug; 44 % of combinations were valproate and lamotrigine.

Major malformations, at 1 year of age, were seen in 10 % of children exposed to valproate alone, in 11.3 % exposed to valproate and lamotrigine and in 11.7 % exposed to valproate and another anti-epileptic drug that was not lamotrigine. Their major finding was that it was the dose of valproate that predicted malformation rate with doses of over 1.5 g a day associated with a major increase: 24 % monotherapy; 31 % valproate and lamotrigine; 19.2 % valproate and another drug.

Comment: This is further evidence that the dose of valproate matters, even more than any additional anti-epileptic medication it may be taken with. Studies such as this are important and laudable. However, by cleaning the data by removing women with other concomitant drugs and previous health problems it can be argued that these data remain difficult to interpret in a real-world situation. Anti-epileptic drugs are notorious for their drug–drug interactions and valproate and lamotrigine interactions are clinically relevant and may underlie the large proportion malformations when a large valproate dose is prescribed together with lamotrigine. EURAP, in keeping with many studies, struggles to accurately classify epilepsy within this cohort and, therefore, diagnosis-specific effects remain unclear.

Tomson T et al (2015) Neurology 85(10):866–872.

## Prospective assessment of autism traits in children exposed to anti-epileptic drugs during pregnancy

Anecdotal reports and retrospective analyses of the cognitive outcomes of children exposed to valproate in utero have prompted more detailed epidemiological research. However, for causality to be fully determined, adequately powered prospective studies are needed and initial studies that identified valproate-associated neurodevelopmental disorders including autism, need replicating and verifying. Wood and colleagues identified 105 children aged 6–8 via the Australian Pregnancy Register for Women on Antiepileptic Medication. They used a rating scale to estimate the level of autistic traits in these children and determined that children who scored above a certain cutoff displayed ‘autistic traits’.

Valproate exposure, and in particular higher doses of valproate, was significantly associated with autistic traits in the children studied. 10.5 % demonstrated autistic traits—of these, all bar two were exposed to valproate, and 64 % were taking valproate as part of a polytherapy regimen. In total, 47 % of women who took valproate alongside one or more anti-epilepsy drug had children with autistic traits. Further analyses identified that women who did not take folic acid supplements in the first trimester and those who smoked marijuana, were both more likely to have children who demonstrated autistic traits.

Comment: Children with autistic traits in this study were exposed to valproate doses of between 1 and 3 g/day, mostly in combination with other anti-epilepsy drugs. This study was able to identify and control for factors such as maternal IQ, the type of epilepsy and seizure severity in pregnancy. The sample size and study design were not sufficient for polytherapy combinations to be analysed. In the discussion, the authors report that a number of mothers of children who had high scores on the rating scale, also had further children with autism diagnoses. They postulate that an as yet unproven genetic susceptibility may confer some of the autistic trait risk seen in this study.

Wood AG et al (2015) Epilepsia 56(7):1047–1055.

## Anti-epileptic drugs and intrauterine death: a prospective observational study from EURAP

The pregnancy must come to term for pregnancy outcomes such as malformations and autism to be studied. Most major teratogens also cause intrauterine death. This study was prompted by a Danish registry paper that failed to find a link between anti-epileptic drugs and spontaneous abortion in all women, but did when women with epilepsy were concerned. Using the EURAP registry (described above) the authors identified 7055 eligible pregnancies in 6146 women. There were 592 spontaneous abortions (8.4 %) and 40 stillbirths (0.6 % of pregnancies). No single drug at any dose was associated with an increased risk; however, the rate of intrauterine death was higher in foetuses exposed to polytherapy (12.1 %). Other factors identified would be expected to increase the intrauterine death rate in all cohorts studied: maternal age; parental congenital malformation; prior intrauterine deaths.

Comment: This is a large and well-designed study which supports clinicians in answering difficult and nuanced questions in clinic. The major deficiency in the study is the absence of a marker of epilepsy severity and the ability to quantify the number of convulsive seizures that each mother had in pregnancy. Furthermore, the EURAP registry was unable to answer an important related question—what is the risk to women with epilepsy and children who are unmedicated in pregnancy? This is important as current guidance is unanimous in stating that the risks of seizures in pregnancy are greater than the risks of the drugs, and that pregnant women should remain on medication. Although whether this is the case for all women, for all epilepsies, and all drugs remains unclear. What is more certain is that when taken as a trio of papers it becomes clear that when women of reproductive age require anti-seizure medication, we should prescribe monotherapy; and if it has to be valproate, then it must be the smallest dose possible.

Tomson T et al (2015) Neurology 85(7):580–588.

